# CircRILPL1 promotes muscle proliferation and differentiation via binding miR-145 to activate IGF1R/PI3K/AKT pathway

**DOI:** 10.1038/s41419-021-03419-y

**Published:** 2021-02-01

**Authors:** Xuemei Shen, Jia Tang, Rui Jiang, Xiaogang Wang, Zhaoxin Yang, Yongzhen Huang, Xianyong Lan, Chuzhao Lei, Hong Chen

**Affiliations:** Key laboratory of Animal Genetics, Breeding and Reproduction of Shaanxi Province, College of Animal Science and Technology, Northwest A&F University, Yangling, Shaanxi China

**Keywords:** Cell growth, Cell proliferation

## Abstract

Many novel non-coding RNAs, such as microRNAs (miRNAs) and circular RNAs (circRNAs), are involved in various physiological and pathological processes. The PI3K/AKT signaling pathway is important for its role in regulating skeletal muscle development. In this study, molecular and biochemical assays were used to confirm the role of miRNA-145 (miR-145) in myoblast proliferation and apoptosis. Based on sequencing data and bioinformatics analysis, we identified a new circRILPL1, which acts as a sponge for miR-145. The interactions between circRILPL1 and miR-145 were examined by bioinformatics, a luciferase assay, and RNA immunoprecipitation. Mechanistically, knockdown or exogenous expression of circRILPL1 in the primary myoblasts was performed to prove the functional significance of circRILPL1. We investigated the inhibitory effect of miR-145 on myoblast proliferation by targeting *IGF1R* to regulate the PI3K/AKT signaling pathway. A novel circRILPL1 was identified that could sponge miR-145 and is related to *AKT* activation. In addition, circRILPL1 was positively correlated with muscle proliferation and differentiation in vitro and could inhibit cell apoptosis. The newly identified circRILPL1 functions as a miR-145 sponge to regulate the *IGF1R* gene and rescue the inhibitory effect of miR-145 on the PI3K/AKT signaling pathway, thereby promoting myoblast growth.

## Introduction

Skeletal muscle development is a complex and strict regulatory process that involves the proliferation and differentiation of myoblasts and fusion into multinucleated muscle fibers with contractile properties^1–4^. For humans, muscle development disorders usually cause muscle diseases before birth, such as congenital muscular dystrophy and myasthenic syndrome^5,6^. For livestock, the development of muscle directly impacts the meat yield and commercial value. There is an increasing need for detailed explorations to guide the disease diagnosis and livestock production.

Biotechnology and next-generation sequencing technology have ushered in a new era in molecular diagnosis of muscle diseases and the discovery of developmental genes. For instance, insulin-like growth factor-1 receptor (*IGF1R*) has been reported to effectively induce myoblast proliferation and differentiation via the phosphoinositide 3’-kinase (PI3K)/AKT pathway^7,8^. *IGF1R* plays a key role in transmitting signals from insulin and insulin-like growth factor-1 to the intracellular pathway^9^. In this study, combined with previous research reports, a schematic diagram of the area downstream of this pathway was constructed (Supplementary Fig. 1). The discovery of new functional genes will improve the diagnostics of these malignant muscle diseases and identify new molecular pathways that may serve as potential therapeutic targets.

Furthermore, recent reports have suggested that multiple genes are involved in the complex regulatory network, and non-coding RNAs also play an important role in muscle development^10–12^. For instance, miR-432 can target *E2F3* and *P55PIK* to inhibit myogenesis through the PI3K/AKT signaling pathway^13^. In addition, miR-21 is involved in skeletal muscle development and regulates PI3K/AKT signaling by targeting *TGFβ1*^14^. Studies of other cell types indicate that miR-145 can inhibit the function of *IGF1R*. In endometrial cells, miR-145 can inhibit *IGF1R* gene expression, thereby affecting the implantation of embryos^15^. MiR-143 and miR-145 can regulate *IGF1R* to suppress cell proliferation in colorectal cancer^16^. However, the targeting relationship between miR-145 and *IGF1R* in bovine muscle cells has received little attention.

Recently, circular RNAs (circRNAs), as a kind of endogenous regulatory RNA, have attracted a wide range of research interest. CircRNAs are characterized by covalently closed-loop structures with neither 5′–3′ polarity nor a polyadenylated tail. Many of these highly stable circRNAs are abundantly expressed and play a role in many diseases, especially in tumors, by acting as microRNA (miRNA) sponges, decoying proteins, and affecting translation. For example, CDR1as and circHIPK3 have been fully investigated in other studies on competitive endogenous RNA (ceRNA) networks and variant pathways^17^. CircNRIP1 acts as a miR-149-5p sponge to promote gastric cancer progression via the AKT1/mTOR pathway^18^. CircINSR and circFGFR4, as ceRNA, regulate the expression of muscle proliferation and differentiation-related genes by sponging miR-34a and miR-107^19,20^. Despite these findings, further research is needed to understand better the role of circRNAs in bovine muscle growth.

In this study, we found that miR-145 could participate in the regulation of PI3K/AKT signaling pathway by regulating the target gene *IGF1R*. We analyzed the targeting relationship between miR-145 and *IGF1R*, and investigated the effect of miR-145 on muscle cells. Our findings underscore the central function of miR-145 in coordinating a variety of PI3K/AKT signaling genes during muscle development. Notably, in combination with sequencing technology, a new circRNA circRILPL1 was found to adsorb miR-145 to regulate the expression of the *IGF1R* gene.

## Materials and methods

### Tissue and cell lines

All animal experiments and study protocols were approved by the Animal Care Commission of the College of Veterinary Medicine, Northwest A&F University. All the tissue samples from Qinchuan cattle (Bos taurus Qinchuanensis) (*n* = 3) of three development states (embryonic 90 days, neonatal 3 days and adult 24 months) were collected from a local livestock farm in Xi’an, P.R. China.

Primary myoblasts were isolated and cultured from the bovine *Longissimus dorsi* as previously described^21^. HEK293T cells were purchased from the American Type Culture Collection (ATCC) and were tested negative for mycoplasma contamination. Myoblasts were cultured in high-glucose Dulbecco’s modified Eagle’s medium (DMEM, HyClone, USA) supplemented with 20% fetal bovine serum (FBS, Gibco, USA) and 1% penicillin–streptomycin solution (Gibco, USA). Two days after cells reached confluence, DMEM containing 2% horse serum (HyClone, USA) and 1% penicillin–streptomycin solution was used for myogenesis induction. Myotube identification was performed on the 4th day of induction. HEK293T cells were cultured in DMEM with 10% FBS. They were all cultured at 37 °C with 5% CO_2_.

### RNA and gDNA extraction

Total RNA was extracted from cells and tissues using TRIzol reagent (Invitrogen, Carlsbad, CA, USA) according to the manufacturer’s instructions. The gDNA was extracted using a genomic DNA isolation kit (Sangon Biotech, Shanghai, China). The nuclear and cytoplasmic fractions were extracted using a PARIS kit (Ambion, Life Technologies). RNA was reverse transcribed with a PrimeScript RT reagent kit (Takara, Tokyo, Japan). Real-time qPCR for RNA analyses were performed using SYBR Green PCR Master Mix (Takara, Tokyo, Japan). MiRNAs-specific stem-loop primers were used to perform reverse transcription. Based on the sequence of circRNAs, the divergent primers were designed to determine their authenticity.

### RNase R treatment and actinomycin D assay

Total RNA was incubated for 15 min at 37 °C with or without 5 U/µg RNase R (Epicenter, Madison, WI, USA), and purified by RNeasy MinElute Cleaning kit (Qiagen). Primary myoblasts were exposed to 2 µg/mL actinomycin D (MilliporeSigma, Burlington, MA, USA) at the indicated time point. Total RNA was then extracted to test the half-life of circRILPL1 and linear mRNA.

### Vector construction and cell transfection

The third and fourth exon sequences of the bovine *RILPL1* gene were amplified to construct the pCD2.1-circRILPL1 overexpression vector (Geneseed, Guangzhou, China). The wild-type or the mutant full-length sequence of circRILPL1 was inserted into the *Xhol* I–*Not* I restriction sites of the psiCHECK-2 vector (Promega, Fitchburg, WI, USA). An miR-145 biosensor (psiCHECK-2-miR-145 2×) was created by inserting two copies of an miR-145 reverse complementary sequence in the psiCHECK-2 vector. The predicted 3’-UTR fragment containing the miR-145 binding site in *IGF1R* was amplified by PCR and cloned into the psiCHECK-2 vector. To mutate the presumptive binding site in *IGF1R*, the binding site sequences were replaced as indicated and designated gene-MUT. Small interfering RNA (siRNA) oligonucleotides were designed to combine with the back-splice region. The miR-145 mimics, inhibitors, and corresponding negative control (NC) were synthesized using RiboBio (Guangzhou, China). The mimics and inhibitors (50 nM), siRNA (50 nM), or vectors (2 µg/mL) were transfected into cells with Lipofectamine 2000 (Invitrogen).

### RNA-FISH

Complementary probes (RiboBio, Guangzhou, China) targeting the back-splicing junction region were designed to visualize circRILPL1 fluorescence in situ. Primary myoblasts were first fixed with in situ hybridization fixative. After prehybridization, cells were incubated with the labeled circRILPL1 probes in hybridization buffer at 55 °C overnight. Nuclei were stained with 4′ 6-diamidino-2-phenylindole (DAPI). Laser confocal microscopy was used to observe the localization of circRILPL1 in cells (Nikon, Tokyo, Japan).

### Dual-luciferase reporter assay

HEK293T cells were seeded in 96-well plates for 24 h before transfection. The cells were co-transfected with psiCHECK-2 reporter plasmid, miR-145 mimics, siRNA, or PCD2.1-circRILPL1 vector. The ratio of renilla and firefly luciferase activity was detected with the Dual-Luciferase Reporter Assay Kit (#E2920, Promega, Fitchburg, WI, USA) after 24 h. The optical density of the resulting solution was assessed using the automatic microplate reader (Molecular Devices, Sunnyvale, CA, USA).

### RNA-binding protein immunoprecipitation (RIP)

RIP assay was performed with an EZ-Magna RIP kit (#17-701, Millipore, USA) according to the manufacturer’s instructions. Argonaute2 (Ago2) antibody (Abcam, UK) was used for RIP. The RNA in the immunoprecipitated product was extracted, and the co-precipitated RNA was detected by real-time qPCR after reverse transcription.

### CircRNA pull-down

Biotin-labeled circRILPL1 probe and negative control probe (NC probe) were synthesized by GenePharma (Shanghai, China). We purchased the Pierce™ magnetic RNA-protein pull-down kit (#20164, Thermo, USA) and performed the experiment according to the manufacturer’s instructions. The biotin-labeled probes were incubated with streptavidin magnetic beads for 30 minutes at room temperature. Then, the probe-labeled magnetic beads were incubated with the cell lysates at 4 °C overnight. On the next day, the samples were extracted and eluted with lysis buffer and proteinase K. Finally, real-time qPCR was used to detect the expression levels of circRILPL1 and miR-145 in the immunoprecipitates.

### 5-Ethynyl-2’-deoxyuridine (EdU) and Cell Counting Kit-8 (CCK-8) assay

The cell proliferation was tested by EdU assay kit (RiboBio, Guangzhou, China). After the required transfection, cells were added with 50 µM EdU (5-Ethynyl-2’-deoxyuridine) solution. After incubation for 2 h, proliferating cells were stained with Apollo Dye Solution. Nucleic acids in all cells were stained with Hoechst 33342 (RiboBio, Guangzhou, China). Images were taken using a fluorescence microscope (AMG EVOS, Seattle, WA, USA). We also measured cell proliferation using the Cell Counting Kit-8 (CCK-8, Multisciences, Hangzhou, China) following the manufacturer’s protocols. At 6, 12, 18, and 24 h, the CCK-8 reagent (10 µL) was added to each well and incubated at 37 °C for 2 h. The optical density of CCK-8 at 450 nm was measured using an automatic microplate reader (Synergy4, BioTek, Winooski, USA).

### Cell cycle and apoptosis assay

The cell cycle was assessed using the Cell Cycle Testing Kit (Multisciences, Hangzhou, China). After transfecting for 24 h, cells were harvested and washed with cold phosphate-buffered saline (PBS). Then, the staining solution and permeabilization reagent were added to the cells. After incubating for 30 min, the cell cycle was analyzed by flow cytometry (FACS Canto™ II, BD BioSciences, USA). Myoblasts apoptosis assays were performed with Annexin V-PE/7-AAD Apoptosis Detection Kit (RiboBio, Guangzhou, China) according to the manufacturer’s recommendations. Transfected cells were harvested, resuspended, and incubated with annexin V (5 µL) and 7-AAD (10 µL). Afterward, the apoptosis rate was analyzed using flow cytometry. Flow cytometry analysis was performed on a BD Accuri C6 flow cytometer (FACS Canto™ II, BD Biosciences, USA), and data were processed using FlowJo7.6 software.

### Mitochondrial membrane potential (ΔΨm) assay

To evaluate changes in mitochondrial membrane potential of myoblasts, JC-1 immunofluorescence (Solarbio, Beijing, China) was used based on the instructions. The cells were stained with JC-1 reagent and then analyzed by flow cytometry and fluorescence microscope (AMG EVOS, Seattle, WA, USA).

### Immunofluorescence analysis

After 4 days of induced differentiation, myoblasts were fixed with 4% paraformaldehyde for 30 minutes. After washing with PBS, the cells were incubated at 4 °C overnight with primary anti-MyHC. Finally, the cells were washed and incubated with the corresponding secondary antibody for 2 h. The nuclei were stained with DAPI and observed under a fluorescent microscope. The area of cells labeled with anti-MyHC was measured using Image-pro Plus software. The myotube area was calculated as a percentage of the total image area covered by nuclei.

### Western blot analysis

Proteins from cultured bovine myoblasts were prepared with Radioimmunoprecipitation assay buffer (Solarbio, Beijing, China). Proteins were loaded onto 12% sodium dodecyl sulfate–polyacrylamide gel electrophoresis and transferred onto polyvinylidene difluoride membranes (Thermo Fisher Scientific). The membranes were incubated overnight with primary antibodies specific for anti-GAPDH (1:1000, #ab9485, Abcam, Cambridge, UK), anti-CyclinD1 (CCND1, 1:1000, #ab226977, Abcam, Cambridge, UK), anti-MyoD (1:1000, #ab16148, Abcam, Cambridge, UK), anti-IGF1R (1:500, #bs-0227R, Bioss, Beijing, China), anti-IRS1 (1:500, #bs-0172R, Bioss, Beijing, China), anti-PI3K (1:500, #bs-10657R, Bioss, Beijing, China), anti-Bcl-2 (1:500, #bs-0032R, Bioss, Beijing, China), anti-caspase-9 (1:500, #bs-0049R, Bioss, Beijing, China), anti-Bax (1:500, #bs-0127M, Bioss, Beijing, China), anti-PCNA (1:500, #WL01804, Wanleibio, Haerbin, China), anti-CDK2 (1:500, #WL01543, Wanleibio, Haerbin, China), anti-CyclinE (1:500, #WL01072, Wanleibio, Haerbin, China), anti-AKT (1:500, #WL0003b, Wanleibio, Haerbin, China), anti-p-AKT(Ser473) (1:500, #WLP001a, Wanleibio, Haerbin, China), anti-P53 (1:500, #WL01919, Wanleibio, Haerbin, China), anti-MyoG (1:500, #WL01132, Wanleibio, Haerbin, China), and anti-MyHC (1:500, #WL02785, Wanleibio, Haerbin, China) at 4 °C. The goat anti-mouse IgG (H&L)-horseradish peroxidase (HRP, 1:5000, #bs-40296G, Bioss, China), and goat anti-rabbit IgG (H&L)-HRP (1:5000, #bs-40295G, Bioss, China) were used as secondary antibodies. After incubation with secondary antibodies, the membranes were quantified with the ChemiDoc XRS system (Bio Rad, Hercules, CA, USA).

### Animal studies

C57BL/6 mice were housed in the animal facilities of the Northwest A&F University (China) under conventional conditions. For monitoring muscle regeneration, muscle injury was induced in 6-week-old mice by injecting cardiotoxin (CTX) (50 µL of 10 mM CTX in PBS) into the mid-belly of the right tibialis anterior (TA) muscle. For data reliability, 21 male mice of the same age and weight were selected and randomly divided into seven groups for treatment. No mice died during the test, and all were included in the statistical analysis. CircRILPL1 was injected three times: at 12 h, 48 h, and 96 h after CTX treatment. A total of 6.25 µg of circRILPL1 expression plasmid was prepared by preincubating with Entranster^TM^-in vivo DNA transfection reagent (Engreen Biosystem Co., Ltd.) for 15 min, and injections were made in a final volume of 50 µL in 10% glucose solution. Simultaneously, the other three mice were injected with the same amount of pCD2.1 vector as a control. After CTX treatment (3 d and 6 d), TA muscles were harvested for RNA extraction, and to obtain paraffin sections. Paraffin sections were stained by H&E and immunofluorescence (IF, anti-eMyHC) according to the manufacturer’s instructions.

### Statistical analyses

Data are expressed as the mean ± standard error of at least three independent experiments. Statistical analyses were performed using SPSS 22.0 statistical software (SPSS, Chicago, IL, USA). Student’s *t* test or one-way analysis of variance followed by Bonferroni’s post-test were utilized to determine the significant differences. A probability of 0.05 or less was considered statistically significant.

## Results

### MiR-145 targets IGF1R to inhibit the PI3K/AKT signaling pathway

**Fig. 1:**
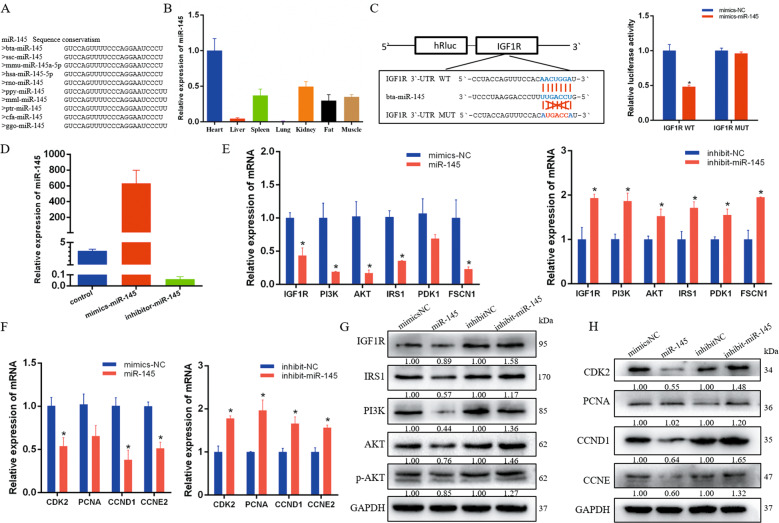
miR-145 targets IGF1R to inhibit the PI3K/AKT signaling pathway in bovine myocytes. **A** Sequence conservation analysis of miR-145 among different species. **B** miR-145 expression pattern in different cattle tissues. **C** The dual fluorescence reporter system verified the targeting relationship between miR-145 and the *IGF1R* gene. **D** Real-time qPCR detected miR-145 overexpression and interference efficiency in bovine primary muscle cells. **E**, **G** The mRNA and protein levels of PI3K/AKT pathway-related genes in bovine myocytes with miR-145 overexpression and inhibition. **F**, **H** The mRNA and protein levels of cell proliferation-related genes in bovine myocytes with miR-145 overexpression and inhibition. *n* = 3. *P < 0.05.

Cattle pre-miR-145 is located on chromosome 7 and is highly conserved among species (Fig. 1A). Real-time qPCR results showed that the expression of miR-145 was high in the calf muscle tissue (Fig. 1B). As predicted by bioinformatics program TargetScan7.1, *IGF1R*, a well-known regulator of the PI3K/AKT pathway, is a potential target of miR-145. The luciferase reporter assay showed that miR-145 mimics significantly inhibited the relative luciferase activity of *IGF1R* WT but did not repress the MUT group (Fig. 1C). To study the function of miR-145 in muscle cells, we chose mimics and inhibitors to overexpress and interfere with miR-145 (Fig. 1D). The results showed that overexpression of miR-145 inhibited the expression of its target gene IGF1R and genes related to the PI3K/AKT signaling pathway. Interference with miR-145 had the opposite effect (Fig. 1E, G). In addition, overexpression of miR-145 significantly inhibited the expression of cell proliferation-related genes at both mRNA and protein levels (Fig. 1F, H). EdU analysis showed that miR-145 mimics significantly decreased the number of myoblasts in the proliferative phase, while interference with miR-145 promoted cell proliferation (Supplementary Fig. 2A). CCK-8 detection showed the same results (Supplementary Fig. 2B, C). In order to investigate whether miR-145 affects the cell cycle, we used flow cytometry to analyze the phase distribution of proliferating myoblasts. These results indicated that the number of cells at G0/G1 increased, whereas those at the S phase decreased upon miR-145 overexpression. The cell cycle change in G2 phase was not significant (Supplementary Fig. 2D, E). The results indicated that miR-145 induced the G0/G1 phase arrest.

### MiR-145 promotes the apoptosis of bovine primary myocytes

**Fig. 2:**
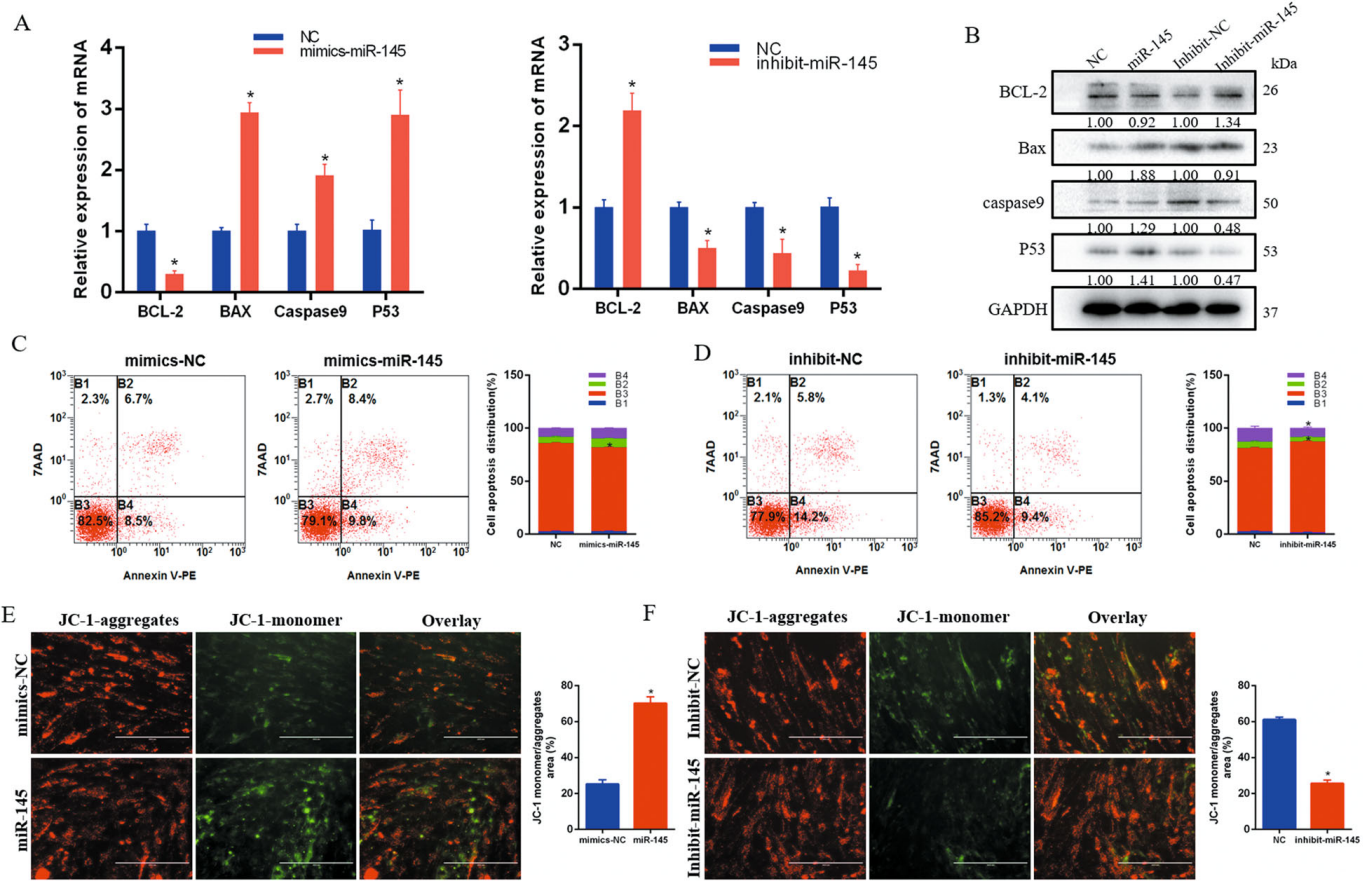
miR-145 promotes the apoptosis of bovine primary myocytes. **A**, **B** The expression of *Bcl-2*, *Bax*, *caspase9*, and *p53* was detected by real-time qPCR and western blots. **C**, **D** Cell apoptosis was determined by Annexin V/7-AAD dual staining followed by flow cytometry. **E**, **F** Cells were stained with JC-1 and images were acquired using a fluorescence microscope. Scale bars, 200 µm. Data are shown as means ± SEM for three individuals. *n* = 3. *P < 0.05.

In investigating the effect of miR-145 on apoptosis, we found lower expression of *Bcl-2* and higher expression of *Bax*, *caspase9*, and *p53* in the miR-145 mimics group than in the NC group. Interference with miR-145 had the opposite effect (Fig. 2A, B). The results of Annexin V-PE/7-AAD staining and flow cytometry showed that the introduction of miR-145 mimics increased the number of apoptotic cells (Fig. 2C), yet interfering with miR-145 could inhibit apoptosis (Fig. 2D).

Decreased mitochondrial membrane potential (ΔΨm) is one of the indicators for cell apoptosis. JC-1 is a fluorescent label widely used to detect ΔΨm, and apoptotic cells emit green fluorescence. Compared with the mimics-NC group, the miR-145 mimics treatment increased green fluorescence, revealing that miR-145 could induce mitochondrial membrane depolarization (Fig. 2E). However, interference with miR-145 can inhibit ΔΨm change (Fig. 2F).

### CircRILPL1 serves as a miR-145 sponge

**Fig. 3:**
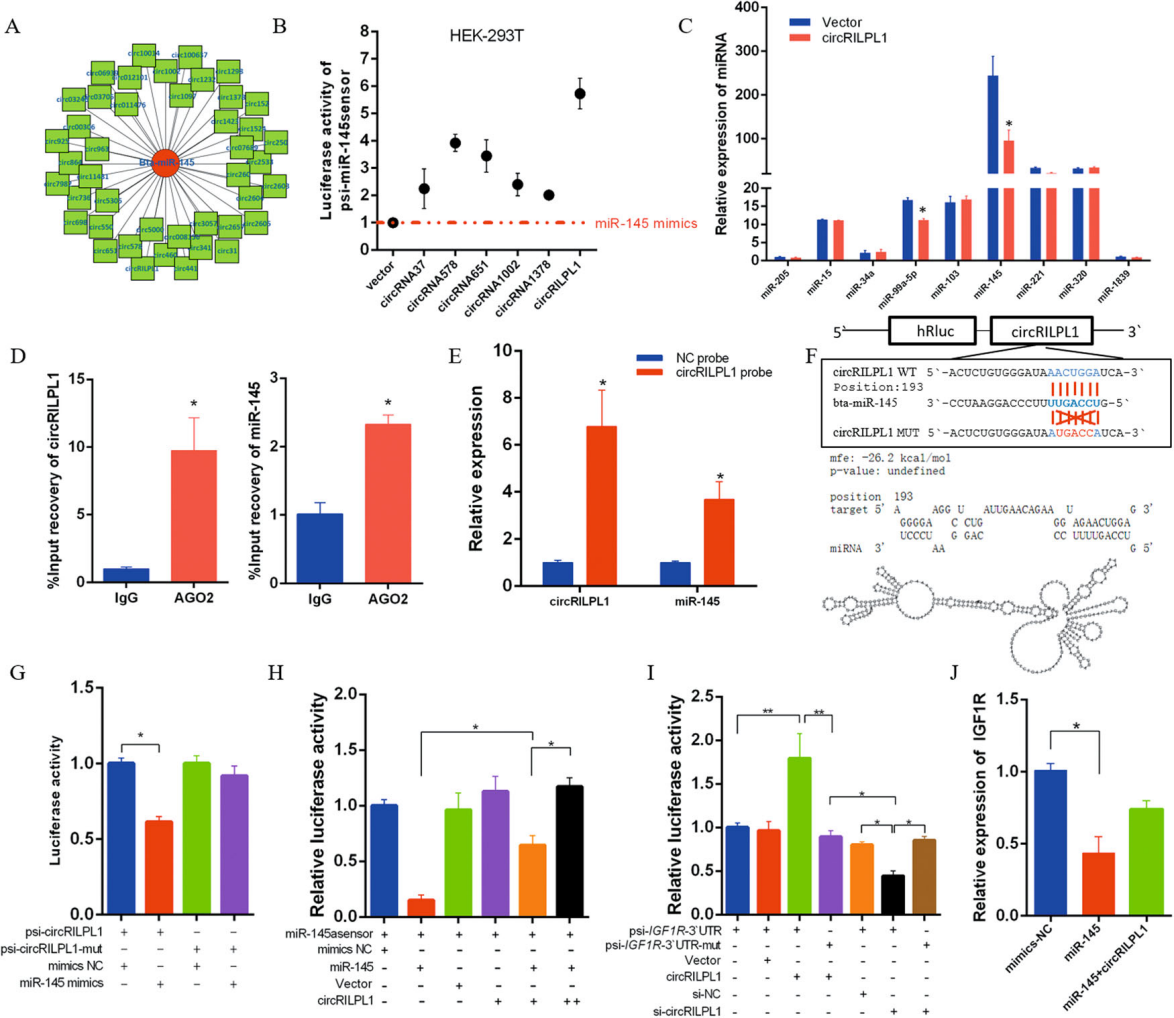
circRILPL1 serves as a miR-145 sponge. **A** CircRNAs with miR-145 absorption sites were screened from high-throughput sequencing data. **B** Luciferase activity of psiCheck2-miR-145 sensor in HEK293T cells co-transfected with miR-145 mimics and circRNAs, which are putatively binding to the miR-145 sequence. Luciferase activity was normalized by firefly luciferase activity. **C** Effect of circRILPL1 on the abundance of miRNAs. **D** Ago2-RIP assay for the amount of circRILPL1 and miR-145 in bovine myoblasts. **E** CircRNA pull-down assays were performed using a specific biotin-labeled circRILPL1 probe in myoblasts. Real-time qPCR was used to detect the expression levels of circRILPL1 and miR-145 in immunoprecipitates. *n* = 3. **P < 0.01. Compared with negative control (NC) probe. **F** Predicted circRILPL1 secondary structure and binding site to miR-145. **G** Luciferase reporter activity of circRILPL1-WT and circRILPL1-mut in HEK293T cells co-transfected with miR-145 mimics or NC mimics. **H** The miR-145 biosensor (psiCHECK-2-miR-145 2×) was transfected into HEK293T cells, together with mimics-NC, miR-145 mimics, pCD2.1-non, or pCD2.1-circRILPL1, and luciferase activities were measured after transfection. **I** Luciferase reporter activity of IGF1R-3’UTR in HEK293T cells with circRILPL1 knockdown or overexpression. **J** IGF1R expression in myocytes transfected with miR-145 mimics alone or co-transfected with circRILPL1. *P < 0.05. **P < 0.01.

An increasing number of research reports indicate that circRNAs can act as miRNAs sponges to regulate downstream targets. Based on the theory of ceRNA, if the sequence of circRNA is exactly complementary to the miRNA seed region, there may be an adsorption relationship. Therefore, we selected 43 circRNAs exactly complementary to the miR-145 seed region from the high-throughput sequencing data of bovine muscle tissue in our published sequencing data (accession ID: GSE87908) (Fig. 3A and Supplementary Table 1). In designing divergent primers for PCR verification and expression analysis, we selected six circRNAs as analysis objects. A repeated motif complementary to the mature sequence of miR-145 was added to the psiCHECK-2 vector, and it became a strong biosensor for miR-145. Accordingly, the miR-145 biosensors and mimics were co-transfected into HEK293T cells, and the miR-145 mimics should dramatically reduce fluorescence activity. However, when miR-145 biosensors, mimics, and circRNAs overexpression vectors are co-transfected, circRNAs can adsorb a part of miR-145, thereby mitigating the inhibitory effect of miR-145 on the fluorescent activity. Among these six circRNAs, circRILPL1 had the most significant effect (Fig. 3B). After overexpressing circRILPL1 in muscle cells, we tested several miRNAs of interest and found that miR-145 expression was reduced (Fig. 3C).

To validate this adsorption, we conducted an Ago2-RIP assay in bovine myoblasts and examined the expression of endogenous circRILPL1 and miR-145 bound to Ago2 protein. The results indicated that circRILPL1 and miR-145 were highly enriched in Ago2 pellets (Fig. 3D). A biotin-labeled circRILPL1 probe was used to conduct RNA pull-down assays to validate this adsorption relationship. After the extraction of RNA from the immunoprecipitation complex, real-time qPCR detected high expression of miR-145 (Fig. 3E). Bioinformatics analysis also showed that the free energy of binding of circRILPL1 to miR-145 is −26.2 kcal/mol (Fig. 3F). The luciferase activity of the psi-circRILPL1^w^ + miR-145 group was significantly lower than that of the control group (Fig. 3G).

In addition, miR-145 overexpression inhibited the luciferase expression of the biosensor, whereas co-transfection of circRILPL1 alleviated the inhibitory effect of miR-145 (Fig. 3H). The luciferase report analysis results for miR-145 biosensors were the same as in Fig. 3B, and the recovery of which exhibited a dose-dependent effect. Furthermore, we found that circRILPL1 overexpression or knockdown could further increase or reduce the luciferase activity of the IGF1R wild-type reporter (Fig. 3I). We also evaluated the expression of IGF1R in myocytes with overexpressing miR-145. The results showed that miR-145 mimics significantly decreased the expression of the IGF1R gene, but it was rescued by circRILPL1 overexpression (Fig. 3J).

### Characteristics of circRILPL1 in myocytes

**Fig. 4:**
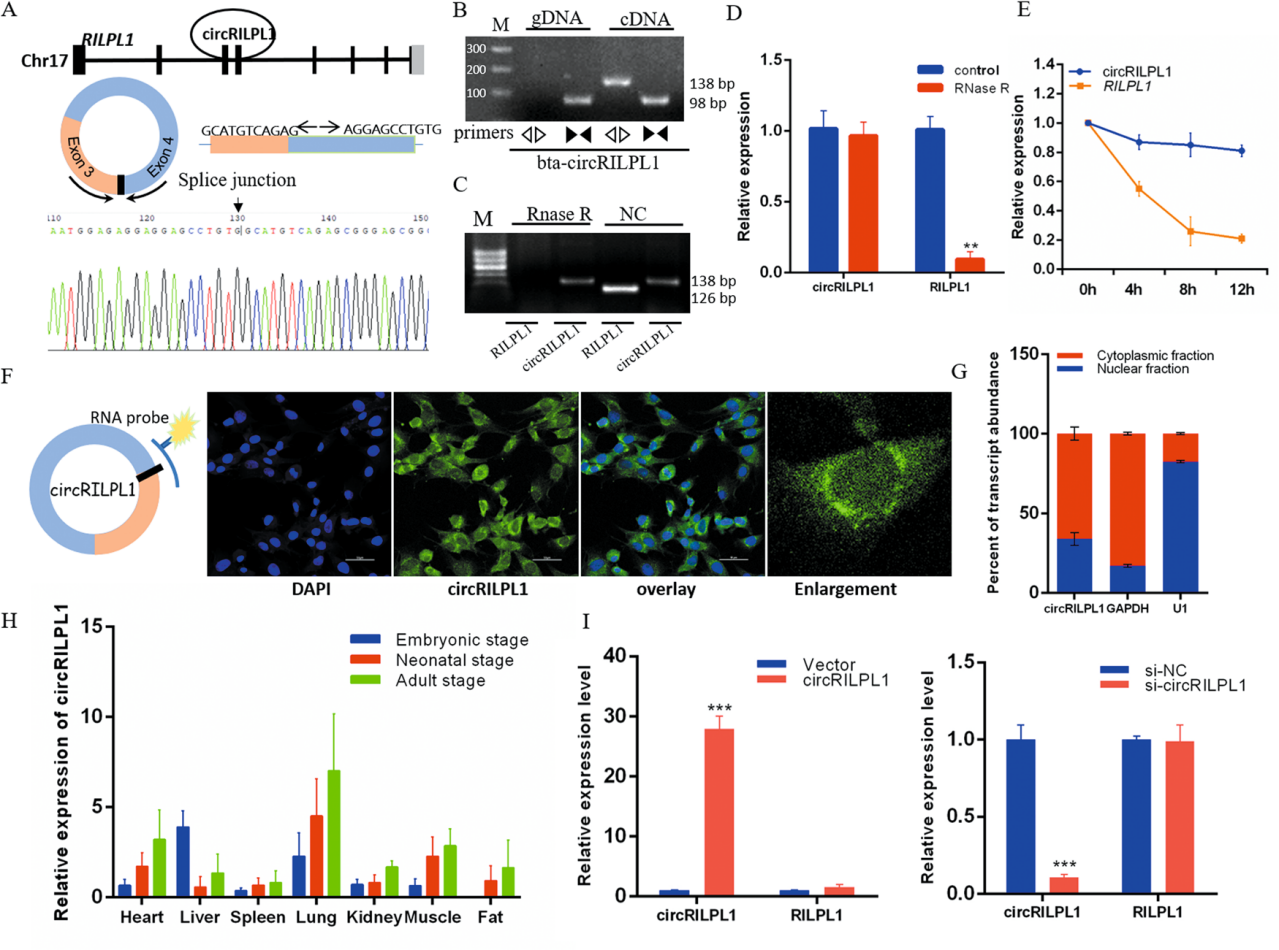
circRILPL1 identification and expression pattern in bovine skeletal muscle. **A** The genomic locus of circRILPL1 (top). The back-splice junction (arrow) of circRILPL1 was identified by Sanger sequencing (bottom). **B** PCR analysis of divergent and convergent primers in cDNA and genomic DNA (gDNA). **C**, **D** Agarose gel electrophoresis and real-time qPCR analysis of circRILPL1 and *RILPL1* levels in myoblasts with and without RNase R treatment. **E** Real-time qPCR for the abundance of circRILPL1 and *RILPL1* in myocytes treated with actinomycin D. **F** FISH detection of circRILPL1 in myoblasts. Scale bars, 50 µm. **G** Levels of circRILPL1 in the nuclear and cytoplasmic fractions of myoblasts. **H** The expression of circRILPL1 in different tissues of cattle at three developmental stages. **I** Real-time qPCR was used to detect the overexpression and interference efficiency of circRILPL1. Data are presented as means ± SEM. *n* = 3. **P < 0.01. ***P < 0.001.

We next analyzed the structure of circRILPL1, which derived from exon 3 to 4 of the *RILPL1* gene (341 nt). Divergent primers were used to amplify the back-spliced junction of circRILPL1 and confirmed by Sanger sequencing (Fig. 4A). PCR analysis showed that the divergent primers could amplify the circular isoform of circRILPL1 with cDNA but no with genomic DNA (gDNA), whereas the convergent primers could produce the linear type from both cDNA and gDNA (Fig. 4B). In myocytes, we detected the stability of circRILPL1 under RNase R and actinomycin D treatment. The results showed that circRILPL1 was resistant to RNase R treatment compared to linear mRNA (Fig. 4C, D), and circRILPL1 was more stable than linear RILPL1 under actinomycin D treatment (Fig. 4E).

To precisely reveal the cellular localization of circRILPL1, we designed an RNA-FISH probe that specifically recognized the back-spliced junction of circRILPL1. FISH analysis was performed in myocytes, and we found that circRILPL1 was localized in the cytoplasm (Fig. 4F). Nuclear and cytoplasmic separation tests showed the same results (Fig. 4G). By analyzing the expression of circRILPL1 in various bovine tissues of three different developmental stages, we found that circRILPL1 was expressed in all seven bovine tissues we selected (Fig. 4H). Real-time qPCR results showed that circular isoform expression changed after overexpression and interference with circRILPL1, but the linear maternal RILPL1 gene did not change (Fig. 4I).

### CircRILPL1 promotes myocytes proliferation by affecting the PI3K/AKT signaling pathway

**Fig. 5:**
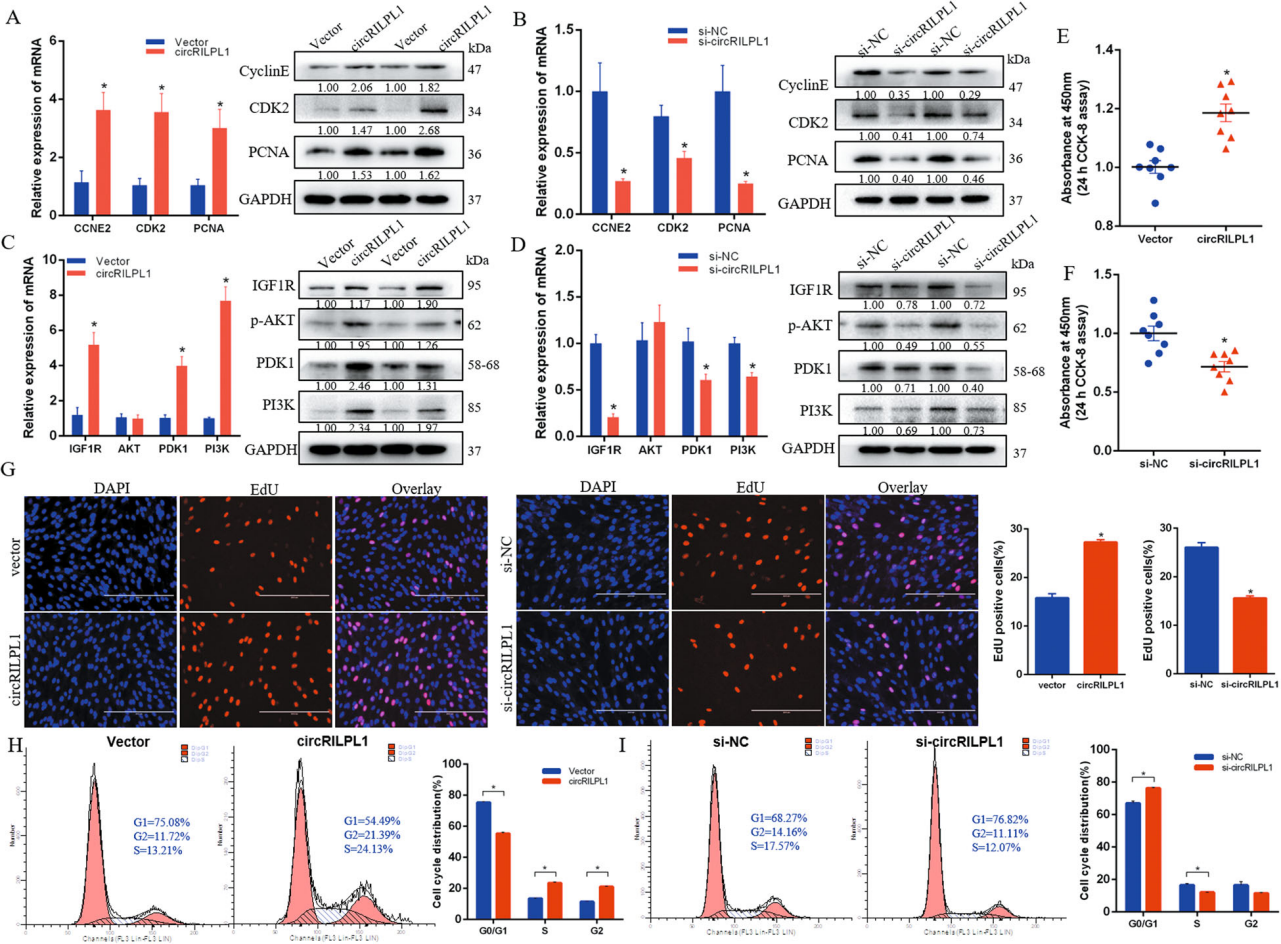
circRILPL1 promotes cell proliferation by affecting the PI3K/AKT signaling pathway. **A**, **B** The expression levels of cell proliferation marker genes were detected by real-time qPCR and western blots. **C**, **D** The expression levels of PI3K/AKT pathway-related genes were detected by real-time qPCR and western blots. **E**, **F** CCK-8 analysis after overexpression and interference with circRILPL1. **G** EdU analysis after overexpression and interference with circRILPL1. Scale bars, 200 µm. **H**, **I** Cell cycle analysis of myocytes with overexpressing or silencing circRILPL1. *P < 0.05.

To explore the physiological functions of circRILPL1, we overexpressed and interfered with circRILPL1 in primary myocytes. The results showed that circRILPL1 could affect the expression of genes related to cell proliferation (Fig. 5A, B). Moreover, overexpression of circRILPL1 promoted the expression of *IGF1R*, a target gene of miR-145, and activated the expression of genes related to the PI3K/AKT signaling pathway (Fig. 5C). Interference with circRILPL1 had the opposite effect (Fig. 5D).

EdU (Fig. 5G) and CCK-8 assays (Fig. 5E), performed to determine the proliferation ability of myocytes, revealed that circRILPL1 overexpression significantly increased the DNA synthesis and cell proliferation rate. However, silencing of circRILPL1 exerted the opposite effect on cell proliferation (Fig. 5F, G). Cell cycle analysis illustrated that overexpression of circRILPL1 increased the number of cells in the S/G2 phase but decreased the number of cells in the G0/G1 phase compared to the controls. Interference with circRILPL1 led to cell cycle arrest from the G0/G1 to the S/G2 phase, which indicated a decrease in cell cycle progression (Fig. 5H, I).

### CircRILPL1 inhibits the apoptosis of bovine primary myocytes

**Fig. 6:**
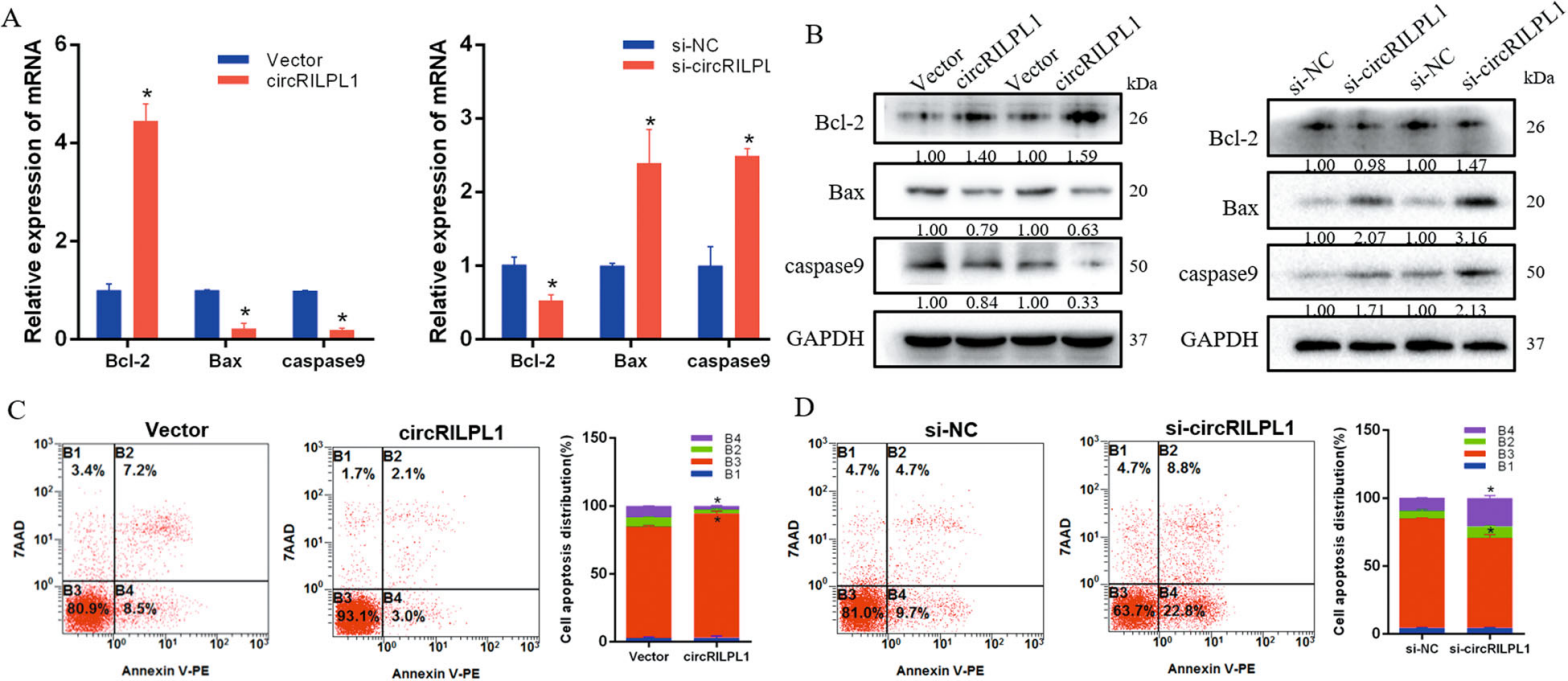
circRILPL1 inhibits the apoptosis of bovine primary myocytes. **A**, **B** The expression of *Bcl-2*, *Bax*, and *caspase9* was detected by real-time qPCR and western blots. **C**, **D** Cell apoptosis was determined by Annexin V/7-AAD dual staining followed by flow cytometry.

To investigate whether circRILPL1 regulates myoblast apoptosis, we used the real-time qPCR and western blotting assays after overexpression or silencing of circRILPL1. The results indicated that overexpression of circRILPL1 could increase the expression of *Bcl-2* and inhibit that of *Bax* and *caspase9*, and interference had the opposite effect. (Fig. 6A, B). We used Annexin V-PE/7-AAD staining to measure apoptosis by flow cytometry. The introduction of siRNA against circRILPL1 resulted in a significant accumulation of apoptotic cells, whereas overexpression decreased (Fig. 6C, D).

### CircRILPL1 facilitates cell proliferation and differentiation by relieving repression of miR-145 for IGF1R expression

**Fig. 7:**
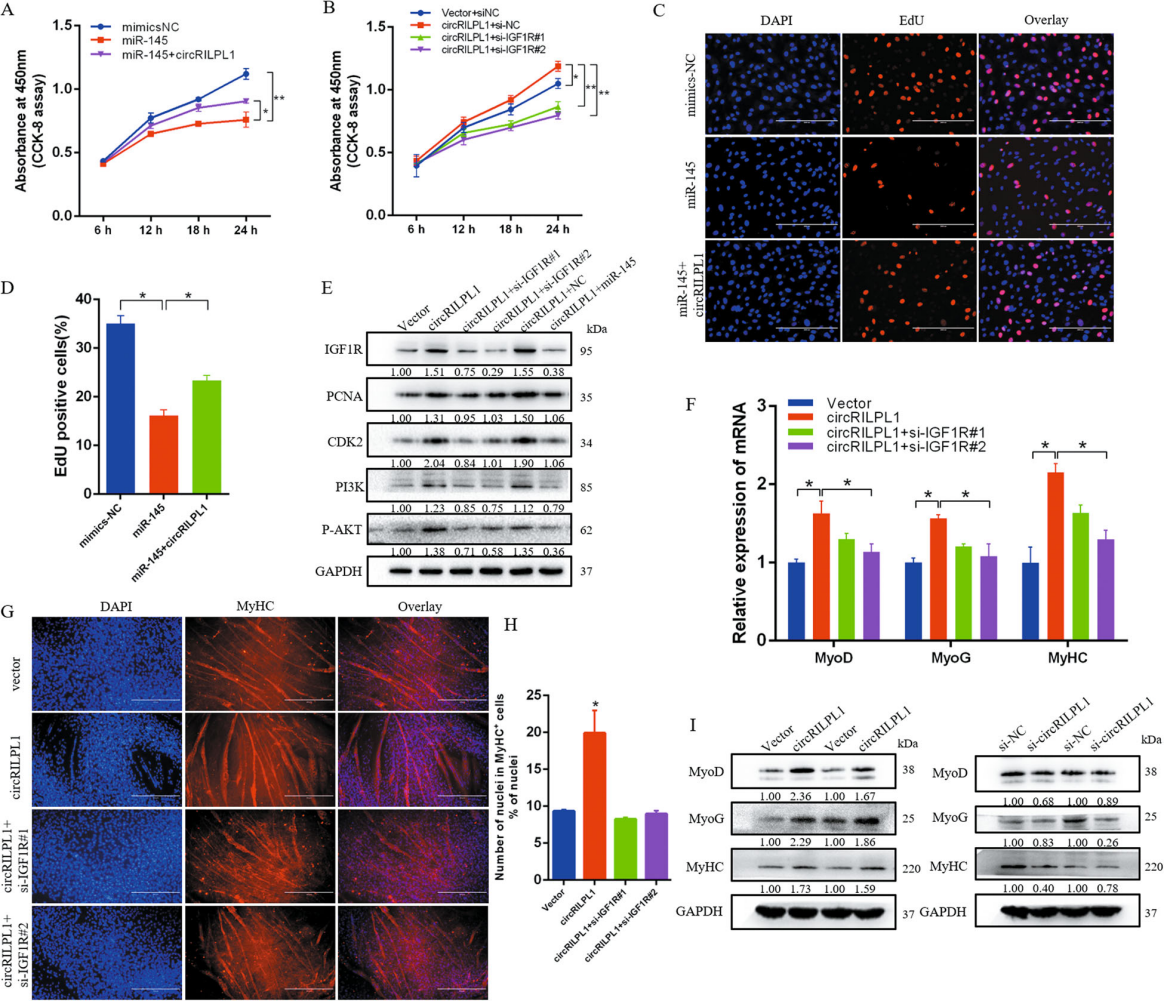
circRILPL1 regulates IGF1R through miR-145. **A** CCK-8 assay for myocytes transfected with miR-145 mimics alone or co-transfected with circRILPL1. *n* = 6. **B** CCK-8 assay for myocytes with circRILPL1 overexpression and knockdown of *IGF1R*. *n* = 6. **C**, **D** EdU assay for myocytes transfected with miR-145 mimics alone or co-transfected with circRILPL1. Scale bars, 200 µm. **E** Cell proliferation and PI3K/AKT pathway-related protein analysis for myocytes transfected with circRILPL1 alone or co-transfected with si-IGF1R and miR-145 mimics. **F** The expression of *MyoD*, *MyoG*, and *MyHC* was detected by real-time qPCR. **G** Myocytes were transfected with circRILPL1 alone or co-transfected with si-IGF1R, immunofluorescence (MyHC) was used to analyze the level of muscle cell differentiation. Scale bars, 500 µm. **H** After 4 days of differentiation, the percentage of nuclei present in MyHC-positive myotubes with the indicated number of nuclei were quantified in cells. Data are presented as mean ± SEM of three independent experiments. **I** The protein expression of MyoD, MyoG, and MyHC was detected by western blots.

To further confirm the cellular phenotype caused by circRILPL1 and miR-145, we co-transfected circRILPL1 with miR-145 mimics or si-IGF1R in myocytes. Cell proliferation analysis showed that overexpression of miR-145 inhibited cell proliferation, and co-transfected circRILPL1 rescued miR-145 mediated suppression for proliferation (Fig. 7A). As with previous results, EdU results indicated that miR-145 mimics significantly inhibited cell proliferation, and co-transfection of circRILPL1 could alleviate this inhibition (Fig. 7C, D). We designed two siRNAs to knockdown the *IGF1R* gene (Supplementary Fig. 5C). The CCK-8 and EdU analysis results showed that knockdown of the *IGF1R* gene could override the effects of circRILPL1 on promoting cell proliferation (Fig. 7B, Supplementary Fig. 5A, B). The results of real-time qPCR and western blots analyses showed that IGF1R siRNAs and miR-145 mimics significantly attenuated the effects of circRILPL1 on *IGF1R*, *PCNA*, *CDK2*, *PI3K*, and *AKT* (Fig. 7E, Supplementary Fig. 4).

In addition, we found that overexpression of circRILPL1 significantly increased the expression of muscle differentiation-related genes (Supplementary Fig. 5D). Furthermore, co-transfection of si-IGF1R significantly attenuated the effects of circRILPL1 on *MyoD*, *MyoG*, and *MyHC* (Fig. 7F). The western blots demonstrated consistent results (Fig. 7I). We propose that circRILPL1 could promote the differentiation of muscle cells through the downstream pathway of *IGF1R*. We analyzed the *MyHC* gene by immunofluorescence. After 4 days of differentiation, the percentage of nuclei present in MyHC-positive myotubes with the indicated number of nuclei were quantified in cells. The results showed that compared with the control group, the number of nuclei in MyHC-positive myotubes increased after overexpression of circRILPL1, indicating that circRILPL1 promoted myocyte accretion during differentiation and led to thickening of myotubes (Fig. 7G, H). Moreover, after interfering with circRILPL1 in the cells, the number of nuclei in MyHC-positive myotubes dropped to about 4%, which was significantly lower than the control group (Supplementary Fig. 5E). However, the differentiation-promoting effect of circRILPL1 would be weakened when si-IGF1R was co-transfected (Fig. 7G, H). However, interfering with IGF1R gene after overexpression of circRILPL1 could significantly reduce the number of nuclei in MyHC-positive myotubes, indicating that the differentiation-promoting effect of circRILPL1 will be weakened when si-IGF1R was co-transfected (Fig. 7G, H).

### Overexpression of circRILPL1 accelerates injury-induced muscle regeneration in vivo

**Fig. 8:**
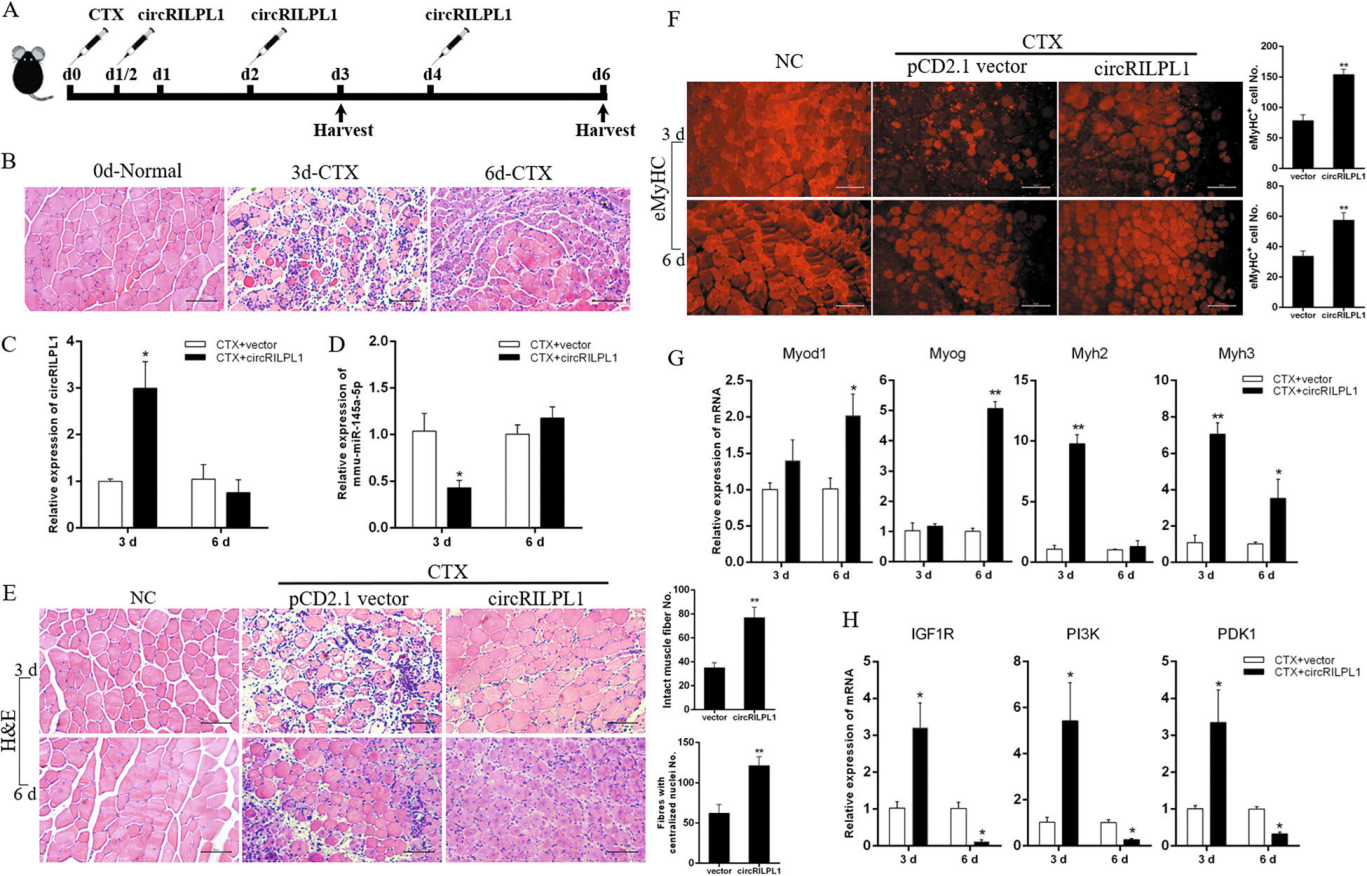
CircRILPL1 improves mouse muscle regeneration in vivo. **A** Injection scheme for circRILPL1 into CTX-injured muscles. *N* = 3 mice for each group. **B** H&E staining of TA muscle at 0, 3, and 6 days after injection of CTX. Scale bars, 100 µm. **C** The expression of circRILPL1 in the above-injected muscles after injection of overexpression plasmid. The data were normalized to GAPDH mRNA. **D** The expression of mmu-miR-145a-5p in the above-injected muscles after injection of circRILPL1 plasmid. The data were normalized to U6. **E** After circRILPL1 expression plasmid injection, muscle H&E staining was performed on the 3rd and 6th day of muscle injury. The empty pCD2.1 vector was injected as a control. Mice in which CTX-muscle injury was not induced served as negative controls (NC). Scale bars, 100 µm. Fibers with intact structure (3 d) or centrally localized nuclei (CLN, 6 d) were quantified. **F** The above fibers were stained for eMyHC. Scale bars, 100 µm. The positively stained fibers were quantified. **G**, **H** The expression of *IGF1R*, *PI3K*, *PDK1*, and myogenic markers in the above-injected muscles. The data were normalized to GAPDH mRNA and represent the average of three independent experiments. Data are presented as means ± SEM. *n* = 3. *P < 0.05. **P < 0.01.

To further verify our findings and avoid the challenges of in vivo experiments in large animals, we used a mouse CTX-induced muscle regeneration model. Following the administration scheme outlined in Fig. 8A, we injected the circRILPL1 plasmid into CTX-injured mouse TA muscle. To verify whether the CTX-induced muscle injury model was successfully established, we collected the muscle tissues at 0, 3, and 6 days after CTX treatment for H&E staining. After three days of CTX treatment, the mice showed extensive myofibers degeneration and vacuolation at the injured site, including many scattered nuclei and blood cells. Six days after the injection of CTX, the partially damaged myofibers in the mice were cleared and replaced by newly formed myofibers containing the central nucleus (Fig. 8B). The results proved that the muscle injury model was successfully constructed, and at 6 days, the injuries had begun to repair the injury.

Results from real-time qPCR analyses showed that the expression of circRILPL1 and miR-145 were overexpressed and decreased, respectively, in the 3-day samples (Fig. 8C, D), suggesting a successful overexpression of circRILPL1 by this approach. However, there was no significant change in the 6-day sample, which may be caused by the replacement of damaged muscles by new muscle cells owing to intense muscle regeneration. In terms of phenotype, as shown in Fig. 8E, overexpression of circRILPL1 after 3 days of muscle injury can significantly reduce the number of broken muscle fibers. At 6 days, only part of the new muscle fibers contained the central nucleus existed in the vector group. However, after overexpression of circRILPL1, all damaged muscle fibers were replaced by central nucleus muscle fibers (Fig. 8E). Consistently, IF staining of the muscle sections revealed an increased number of embryonic MyHC-positive cells (eMyHC, a marker for regenerating fibers) with circRILPL1 injection (Fig. 8F). Expression levels of *MyoD1*, *myogenin*, *myh2*, and *myh3* were found upregulated by circRILPL1 injection (Fig. 8G), suggesting an acceleration of the myogenic process as discovered in vitro. Moreover, with the changes of circRILPL1, the expression of *IGF1R* and AKT pathway-related genes in the 3-day sample increased, then decreased on the 6th day (Fig. 8H). Together, these findings imply that circRILPL1 is a functional myogenic factor during muscle regeneration in vivo.

## Discussion

Skeletal muscle development is regulated by complex regulatory networks composed of coding and non-coding genes. The rapid development of high-throughput sequencing and bioinformatics has led to discovering an increasing number of non-coding RNA, participating in various cellular physiological processes. Emerging evidence indicates that miRNAs and circRNAs play important roles in muscle development. It is crucial to study the molecular regulation mechanisms in the muscle development process because it can improve animal husbandry production, diagnose human disease, and refine molecular therapy.

Recently, the role of the PI3K/AKT signaling pathway in the muscle development regulatory network has become clearer.

Akt inhibits FoxO family transcription factors through phosphorylation, thereby inhibiting muscle cell protein degradation. It also stimulates protein synthesis through the mammalian targets of rapamycin (mTOR) and glycogen synthase kinase 3b (GSK3b)^22^. Furthermore, Akt can act on CDK inhibitors P21 and P27 and indirectly affect the expression level of *CyclinD1* and *p53* to regulate cell cycle and cell proliferation^23–25^. Akt also promotes cell survival by directly inhibiting pro-apoptotic signaling factors such as bad and forkhead transcription factors^26,27^. However, activation of the PI3K/AKT signaling pathway requires the participation of *IGF1R*, a heterotetramer consisting of two extracellular ligand-bound alpha chains (IGF1Ra) and two membrane-spanning beta (IGF1Rb) chains that are bound together by disulfide bonds^28^. The binding of IGF1 and IGF1R leads to activation of its intrinsic tyrosine kinase and autophosphorylation, which phosphorylates insulin receptor substrate (IRS), thereby activating downstream PI3K^29^. We can conclude that the IGF1-PI3K/AKT pathway is a key intracellular signaling mechanism controlling muscle development^22^.

Previous studies have shown that many miRNAs are involved in the PI3K/AKT signaling pathway. For example, miR-145, by targeting *IGF1R*, could regulate the proliferation of various cancer cells, including colon cancer^30^, hepatocellular carcinoma^31^, and breast cancer^32^. Consistent with previous studies, our research results showed that miR-145 could regulate the expression of *IRS1, PI3K, FSCN1*, and other genes and ultimately affect the function of the PI3K/AKT signaling pathway^31,33^. In this study, we determined that miR-145 is abundantly expressed in bovine muscle cells and has binding sites in the IGF1R-3′UTR region. Our results indicated that after miR-145 overexpression, the PI3K/AKT signaling pathway and cell proliferation were inhibited (Supplementary Fig. 2) and apoptosis was induced (Fig. 2).

To further study the role of non-coding RNAs in the IGF1R/PI3K/AKT signaling pathway, we focused on circRNAs that adsorb miR-145. Most previous research has searched for downstream miRNAs after studying circRNAs, and we looked for unknown upstream circRNAs after investigating a known miRNA (miR-145). This method informs the future study of circRNAs. In this study, we used existing high-throughput sequencing data combined with bioinformatics analysis techniques to predict 42 circRNAs capable of adsorbing miR-145. We ligated a repetitive miR-145 reverse complementary sequence into the psiCHECK-2 vector, which can precisely sense changes in the amount of miR-145 in cells. The dual-luciferase reporting system results indicated that six circRNAs could rescue the inhibitory effect of miR-145 mimics on Renilla fluorescence. Among them, the effect of circRILPL1 was the most obvious.

circRILPL1 is derived from the third and fourth exons of Rab-interacting lysosomal protein-like 1(*RILPL1*). Combining bioinformatics and expression level analysis, we initially speculated that circRILPL1 has a relationship with miR-145. Dual-luciferase reporter assays, RNA pull-down, and anti-Ago2 RNA immunoprecipitation confirmed that circRILPL1 directly interacts with miR-145. Like all known circRNAs, the newly discovered circRILPL1 has more stable properties than linear RNA under conditions of Rnase R and actinomycin D treatment. In addition, the FISH results showed that the high distribution of circRILPL1 in the cytoplasm also guarantees its adsorption of miRNAs. The functional gain and loss experiments demonstrated that circRILPL1 was associated with the cell cycle, proliferation, and apoptosis. After overexpression of circRILPL1 in muscle primary cells by circRNA-specific vectors, we detected a significant increase in the expression of IGF1R, which is the target gene of miR-145. In addition, AKT pathway-related genes were also significantly overexpressed (Fig. 5). To further verify whether circRILPL1 could function by miR-145, we co-transfected circRILPL1 and si-IGF1R in bovine primary myocytes. We found that overexpression of circRILPL1 and then interference with *IGF1R* may reverse the role of circRILPL1 in activating the PI3K/AKT signaling pathway. Results of the co-transfection experiments also showed that circRILPL1 could abolish the endogenous suppressive effect of miR-145 on the target gene *IGF1R* and participate in the PI3K/AKT signaling pathway.

In addition, previous research indicates that the PI3K/AKT signaling pathway could also affect cell differentiation. In this study, we performed immunofluorescence staining to study the differentiation status of myoblasts using myosin heavy chain (MyHC) antibody, which is a marker of myogenic differentiation. To quantify myoblast fusion, we calculated the fusion index by expressing the number of nuclei within MyHC-positive myotubes with more than two nuclei as a percentage of the total nuclei. Using anti-MyHC for IF staining of the differentiated myocytes, we could see that the myotubes formed by the fusion of myoblasts were stained red. After overexpression of circRILPL1, the number of nuclei in MyHC-positive myotubes was significantly increased, but this differentiation-promoting effect would be inhibited after interference with the *IGF1R* gene. To further examine the functions of circRILPL1 during myogenesis in vivo, we employed a widely used muscle regeneration model, in which the intramuscular injection of CTX could cause muscle injury and, in turn, result in muscle regeneration. We found that circRILPL1 promotes muscle regeneration in mice with TA muscle injury. Therefore, we propose that the circRILPL1 plasmid could adsorb miR-145 in mouse muscles to regulate downstream pathways, thereby promoting muscle regeneration. However, we recognize one shortcoming of our study: the miR-145 and IGF1R-PI3K/AKT pathway we explored may only be one of the pathways through which circRILPL1 functions. circRILPL1 may also be related to other miRNAs or pathways, which require further study.

In summary, we identified a novel circRILPL1 as a sponge for miR-145 to activate IGF1R/PI3K/AKT signaling, thus promoting muscle proliferation, increasing cell differentiation, and suppressing muscle apoptosis (Supplementary Fig. 3). From a medical perspective, as circRILPL1 is closely related to cell proliferation and apoptosis, it may be used as a potential therapeutic agent for treating muscle atrophy. For livestock, our research expands the understanding of non-coding RNAs related to bovine muscle development. circRILPL1 could be applied to molecular marker-assisted breeding.

## Supplementary information

Supplementary information
