# Supplementary material for: CircRILPL1 promotes muscle proliferation and differentiation via binding miR-145 to activate IGF1R/PI3K/AKT pathway
Source: Cell Death Dis. 2021 Feb 1;12(2):142. doi: 10.1038/s41419-021-03419-y (PMC7862392; doi:10.1038/s41419-021-03419-y)
Supplement: Supplementary file 1 — Supplementary information [file 41419_2021_3419_MOESM1_ESM.docx]

**Supplementary Information**

circRILPL1 promotes muscle proliferation and differentiation via binding miR-145 to activate IGF1R/PI3K/AKT pathway

**Shen et al.**

**Supplementary Table 1.**

Predicted 43 circRNAs binding to miR-145

| **circRNA** | **Chr** | **start** | **end** | **GeneName** | **Spliced length** |
| --- | --- | --- | --- | --- | --- |
| circRNA37 | chrX | 79761001 | 79786582 | ATRX | 721 |
| circRNA152 | chr13 | 61903418 | 61908701 | MYLK2 | 648 |
| circRNA250 | chr12 | 51190589 | 51192931 | LMO7 | 1002 |
| circRNA260 | chr12 | 79483030 | 79483260 | STK24 | 231 |
| circRNA341 | chr11 | 37588332 | 37591912 | RTN4 | 503 |
| circRNA441 | chr10 | 10368438 | 10373276 | HOMER1 | 423 |
| circRNA460 | chr10 | 18734376 | 18768889 | MYO9A | 689 |
| circRNA550 | chr10 | 102086392 | 102147781 | FOXN3 | 694 |
| circRNA578 | chr17 | 12952841 | 12953417 | SMAD1 | 577 |
| circRNA606 | chr17 | 54374596 | 54375599 | RILPL1 | 341 |
| circRNA651 | chr16 | 26885542 | 26900286 | AIDA | 350 |
| circRNA698 | chr16 | 58794470 | 58795318 | RFWD2 | 197 |
| circRNA736 | chr15 | 17230512 | 17289984 | ALKBH8 | 920 |
| circRNA864 | chr14 | 70317892 | 70318491 | MTERFD1 | 220 |
| circRNA925 | chr19 | 23678980 | 23683008 | SMG6 | 324 |
| circRNA963 | chr19 | 46863216 | 46873840 | CDC27 | 1177 |
| circRNA1002 | chr18 | 15963492 | 15984525 | PHKB | 515 |
| circRNA1097 | chr26 | 43074082 | 43084914 | ENSBTAG00000023846 | 939 |
| circRNA1232 | chr25 | 10632803 | 10633196 | GSPT1 | 296 |
| circRNA1298 | chr22 | 9674711 | 9686286 | ARPP21 | 898 |
| circRNA1378 | chr23 | 16344530 | 16345293 | UBR2 | 370 |
| circRNA1423 | chr20 | 32768837 | 32818175 | OXCT1 | 606 |
| circRNA1524 | chr21 | 55313628 | 55318327 | PRPF39 | 1020 |
| circRNA2533 | chr1 | 87976414 | 87979067 | USP13 | 311 |
| circRNA2603 | chr1 | 157203169 | 157242275 | SATB1 | 1364 |
| circRNA2604 | chr1 | 157203169 | 157246201 | SATB1 | 1599 |
| circRNA2605 | chr1 | 157218881 | 157246201 | SATB1 | 1230 |
| circRNA2657 | chr9 | 68178495 | 68178737 | LAMA2 | 243 |
| circRNA3057 | chr17 | 17395881 | 17398919 | TBC1D9 | 699 |
| circRNA5000 | chr5 | 98078528 | 98102610 | LRP6 | 1105 |
| circRNA5305 | chr1 | 87974292 | 88033588 | USP13 | 2058 |
| circRNA7982 | chr5 | 68262848 | 68295501 | TXNRD1 | 1467 |
| circRNA10014 | chr24 | 34859163 | 34883866 | MIB1 | 1250 |
| circRNA10637 | chr3 | 50463386 | 50463569 | TMED5 | 184 |
| circ_0000306 | chr10 | 45609922 | 45610796 | ZNF609 | 874 |
| circ_0003246 | chr16 | 24216869 | 24218695 | EPRS | 1826 |
| circ_0003705 | chr17 | 12952840 | 12953417 | SMAD1 | 577 |
| circ_0006939 | chr23 | 20081399 | 20082808 | ADGRF5 | 1409 |
| circ_0007689 | chr25 | 28371054 | 28372000 | TPST1 | 946 |
| circ_0008156 | chr27 | 26305612 | 26306033 | WRN | 421 |
| circ_0011476 | chr5 | 98016630 | 98017024 | LRP6 | 394 |
| circ_0011481 | chr5 | 98100985 | 98102610 | LRP6 | 1625 |
| circ_0012101 | chr7 | 13657459 | 13657991 | NFIX | 532 |

**Sequence of circRILPL1**

>circRILPL1

GCATGTCAGAGCGGGAGCGGCAGGTGATGAAGAAGCTGAAGGAGGTGGTGGACAAACAGCGAGATGAGATCCGTGCCAAAGATCGGGAGCTGGGGCTGAAGAACGAGGATGTTGAGGCGCTGCAGCAGCAGCAGACCCGGCTGATGAAGATCAACCACGACCTCCGGCATCGAGTCACAGTGGTAGAGGCCCAGGGGAAGGCTCTGATTGAACAGAAGGTAGAACTGGAGGCAGATCTGCAAACCAAGGAGCAGGAGATGGGCAGCCTGCGGGCGGAACTTGGGAAGCTGCGAGAGAGGCTACAAGGCGAACTCAACCAAAATGGAGAGGAGGAGCCTGTG

**Supplementary Figure.**


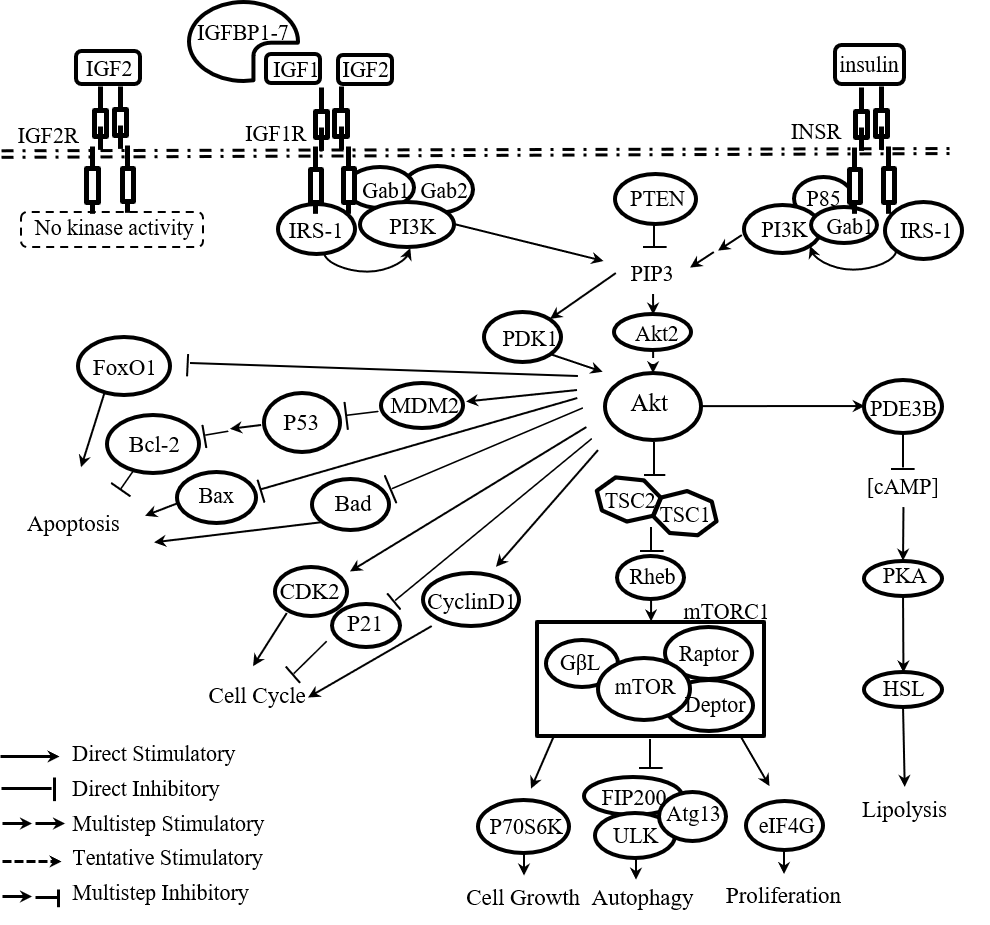


**Supplementary Figure 1.** Schematic diagram of IGF1R transmitting extracellular signals and activating intracellular signaling pathways.

**
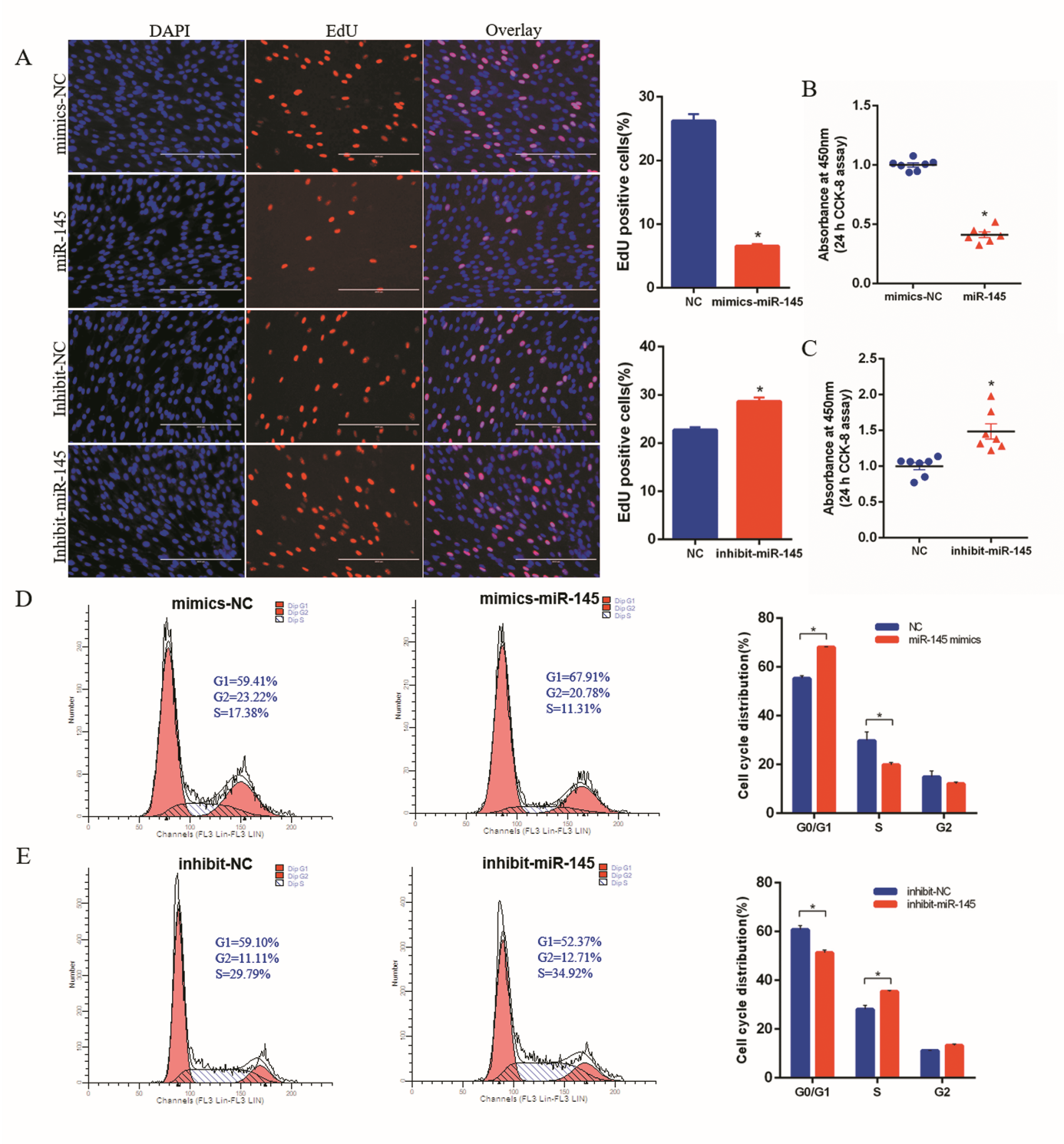
Supplementary Figure 2.** **miR-145 inhibits the** **proliferation of bovine primary myocytes.** (A) Bovine primary myocytes were transfected with miR-145 mimics and inhibitors, and cell proliferation was analyzed using EdU. Scale bars, 200 µm. n = 3. (B, C) Cell proliferation analysis (24 h) of myocytes with silencing or overexpressing miR-145. n = 6. (D, E) Cell cycle analysis of myocytes with silencing or overexpressing miR-145. Data are presented as means ± SEM. n = 3. **P* < 0.05.


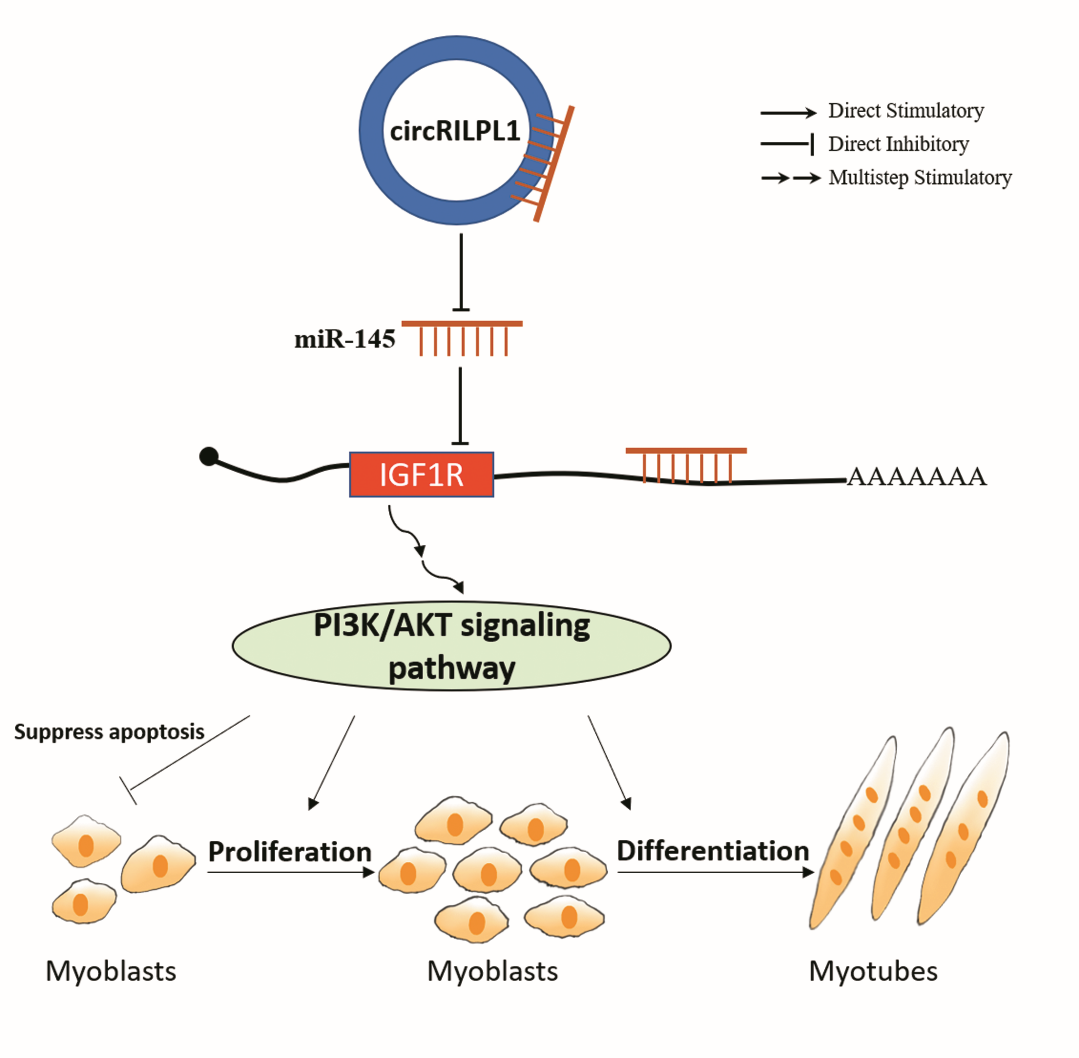


**Supplementary Figure 3. Molecular mechanism of circRILPL1 promotes myoblast proliferation, differentiation, and suppress apoptosis.**

**
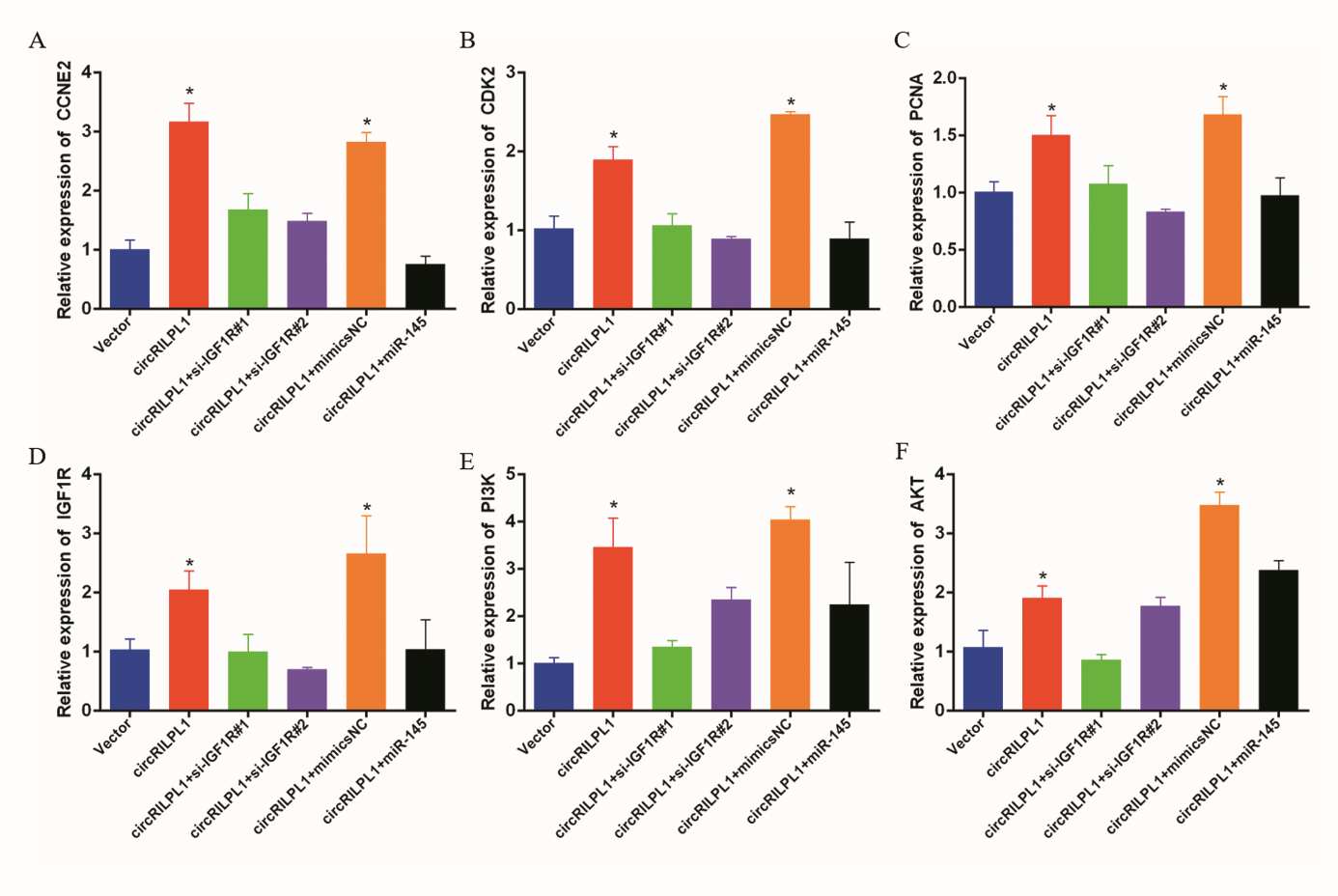
Supplementary Figure 4.** (A-F) Cell proliferation and PI3K/AKT pathway related genes analysis for myocytes transfected with circRILPL1 alone or co-transfected with si-IGF1R and miR-145 mimics.

**
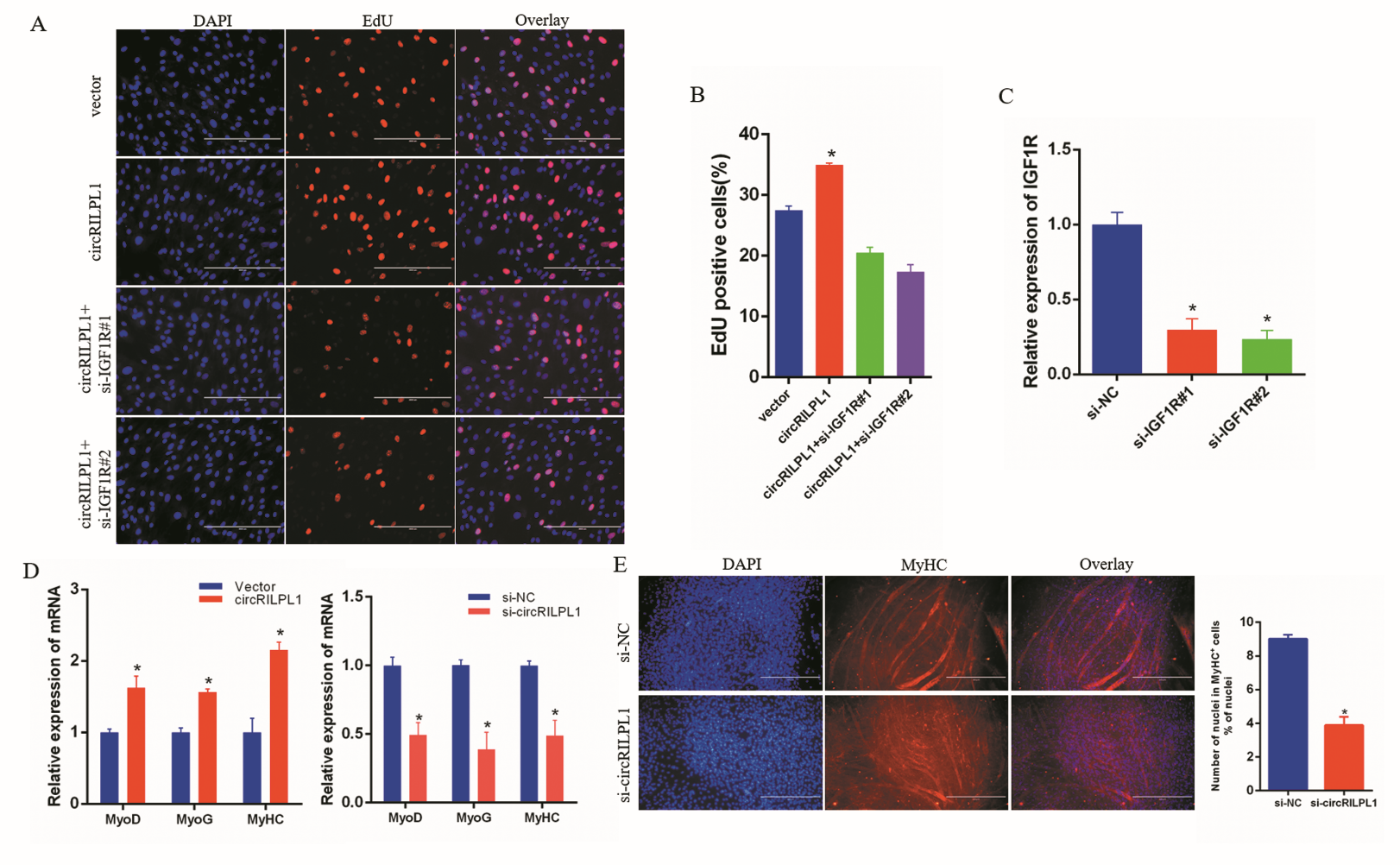
Supplementary Figure 5.** (A, B) EdU assay for myocytes transfected with circRILPL1 alone or co-transfected with si-IGF1R. Scale bars, 200 µm. (C) Interference efficiency of si-IGF1R#1 and si-IGF1R#2 in bovine primary myocytes. (D) The expression of *MyoD*, *MyoG*, and *MyHC* was detected by real-time qPCR. (E) Myocytes were transfected with si-circRILPL1 and si-NC, immunofluorescence (MyHC) was used to analyze the level of muscle cell differentiation. Scale bars, 500 µm. After 4 days of differentiation, the percentage of nuclei present in MyHC-positive myotubes with the indicated number of nuclei were quantified in cells. Data are presented as mean ± SEM of three independent experiments. **P* < 0.05.
